# Correction: Respiratory infection‑ and asthma‑prone, low vaccine responder children demonstrate distinct mononuclear cell DNA methylation pathways

**DOI:** 10.1186/s13148-025-01848-6

**Published:** 2025-03-28

**Authors:** David Martino, Nikki Schultz, Ravinder Kaur, Simon D. van Haren, Nina Kresoje, Annmarie Hoch, Joann Diray‑Arce, Jessica Lasky Su, Ofer Levy, Michael Pichichero, in association with the IDEAL Consortium

**Affiliations:** 1 Wal‑Yan Respiratory Research Centre, Telethon Kids Institute, University of Western Australia, 35 Stirling Highway, Crawley, WA 6009 Australia; 2 Centre for Infectious Disease and Vaccine Immunology, Research Institute, Rochester General Hospital, 1425 Portland Avenue, Rochester, NY 14621 USA; 3 Precision Vaccines Program, Department of Pediatrics, Boston Children’s Hospital, 300 Longwood Ave, BCH 3104, Boston, MA 02115 USA; 4 Channing Division of Network Medicine and Harvard Medical School, Boston, MA 02115 USA; 5 Broad Institute of MIT and Harvard, 415 Main St, Cambridge, MA 02142 USA

**Correction to: Clinical Epigenetics (2024) 16:85** 10.1186/s13148-024-01703-0

The original publication of this article [1] contained an error in the demographic data of Table 1. The incidence of strictly defined otitis media prone (SOP) among cases (infection prone children) and controls (non-infection prone) was incorrectly stated. The published table shows cases of SOP in 10 infection prone children and 0 in non-infection prone but they should have been 11 and 1, respectively.

The incorrect and correct Table 1 have shown with this correction article.

Incorrect Table 1:

**Table 1:** Demographics of study cohort

Variable	Category	IAP	NIAP	Overall	*P*-value
Vaccine responsiveness					0.019
	High vaccine response	(1) 7%	(5) 31%	(6) 20%	
	Normal vaccine response	(6) 43%	(10) 62%	(16) 53%	
	Low vaccine response	(7) 50%	(1) 6%	(8) 26%	
Otitis prone status					< 0.001
	Stringently defined otitis prone	(10) 71%	(0) 0%	(10) 33%	
	Non otitis prone	(4) 29%	(16) 100%	(20) 67%	
Atopy					0.675
	Eczema	(4) 29%	(3) 18%	(7) 23%	
Sex					0.44
	Female	(3) 21%	(6) 38%	(9) 30%	
	Male	(11) 79%	(10) 62%	(21) 70%	
Age (months)	Mean	9.9 ± 2	10.76 ± 1.59	10.36 ± 1.81	0.211
Ancestry					0.485
	African American	(0) 0%	(1) 6%	(1) 3%	
	Caucasian	(14) 100%	(13) 81%	(27) 90%	
	Other	(0) 0%	(2) 12%	(2) 7%	
Daycare					0.142
	Yes	(11) 79%	(8) 50%	(19) 63%	
	No	(3) 21%	(8) 50%	(11) 37%	
Breastfeeding					0.883
	Breastfed	(4) 29%	(3) 19%	(7) 23%	
	Formula fed	(8) 57%	(10) 62%	(18) 60%	
	Both	(2) 14%	(3) 19%	(5) 17%	
Smokers in home					1.00
	Yes	(0) 0%	(1) 6%	(1) 3%	
	No	(14) 100%	(15) 94%	(29) 97%	
Siblings					0.457
	Yes	(7) 50%	(11) 69%	(18) 60%	
	No	(7) 50%	(5) 31%	(12) 40%	

*P*-values are determined using Fishers Exact for categorical variables and t-test for quantitative variables

Correct Table 1:

**Table 1:** Demographics of study cohort

Variable	Category	IAP	NIAP	Overall	*P*-value
Vaccine responsiveness					0.019
	High vaccine response	(1) 7%	(5) 31%	(6) 20%	
	Normal vaccine response	(6) 43%	(10) 62%	(16) 53%	
	Low vaccine response	(7) 50%	(1) 6%	(8) 26%	
Otitis prone status					< 0.001
	Stringently defined otitis prone	(11) 79%	(1) 6%	(12) 40%	
	Non otitis prone	(3) 21%	(15) 94%	(18) 60%	
Atopy					0.675
	Eczema	(4) 29%	(3) 18%	(7) 23%	
Sex					0.44
	Female	(3) 21%	(6) 38%	(9) 30%	
	Male	(11) 79%	(10) 62%	(21) 70%	
Age (months)	Mean	9.9 ± 2	10.76 ± 1.59	10.36 ± 1.81	0.211
Ancestry					0.485
	African American	(0) 0%	(1) 6%	(1) 3%	
	Caucasian	(14) 100%	(13) 81%	(27) 90%	
	Other	(0) 0%	(2) 12%	(2) 7%	
Daycare					0.142
	Yes	(11) 79%	(8) 50%	(19) 63%	
	No	(3) 21%	(8) 50%	(11) 37%	
Breastfeeding					0.883
	Breastfed	(4) 29%	(3) 19%	(7) 23%	
	Formula fed	(8) 57%	(10) 62%	(18) 60%	
	Both	(2) 14%	(3) 19%	(5) 17%	
Smokers in home					1.00
	Yes	(0) 0%	(1) 6%	(1) 3%	
	No	(14) 100%	(15) 94%	(29) 97%	
Siblings					0.457
	Yes	(7) 50%	(11) 69%	(18) 60%	
	No	(7) 50%	(5) 31%	(12) 40%	

*P*-values are determined using Fishers Exact for categorical variables and t-test for quantitative variables
