# Supplementary material for: Respiratory infection- and asthma-prone, low vaccine responder children demonstrate distinct mononuclear cell DNA methylation pathways
Source: Clin Epigenetics. 2024 Jul 3;16:85. doi: 10.1186/s13148-024-01703-0 (PMC11223352; doi:10.1186/s13148-024-01703-0)
Supplement: Supplementary file 1 — Additional file 1. Supplementary Figures S1 and S2. [file 13148_2024_1703_MOESM1_ESM.pptx]

## Slide 1
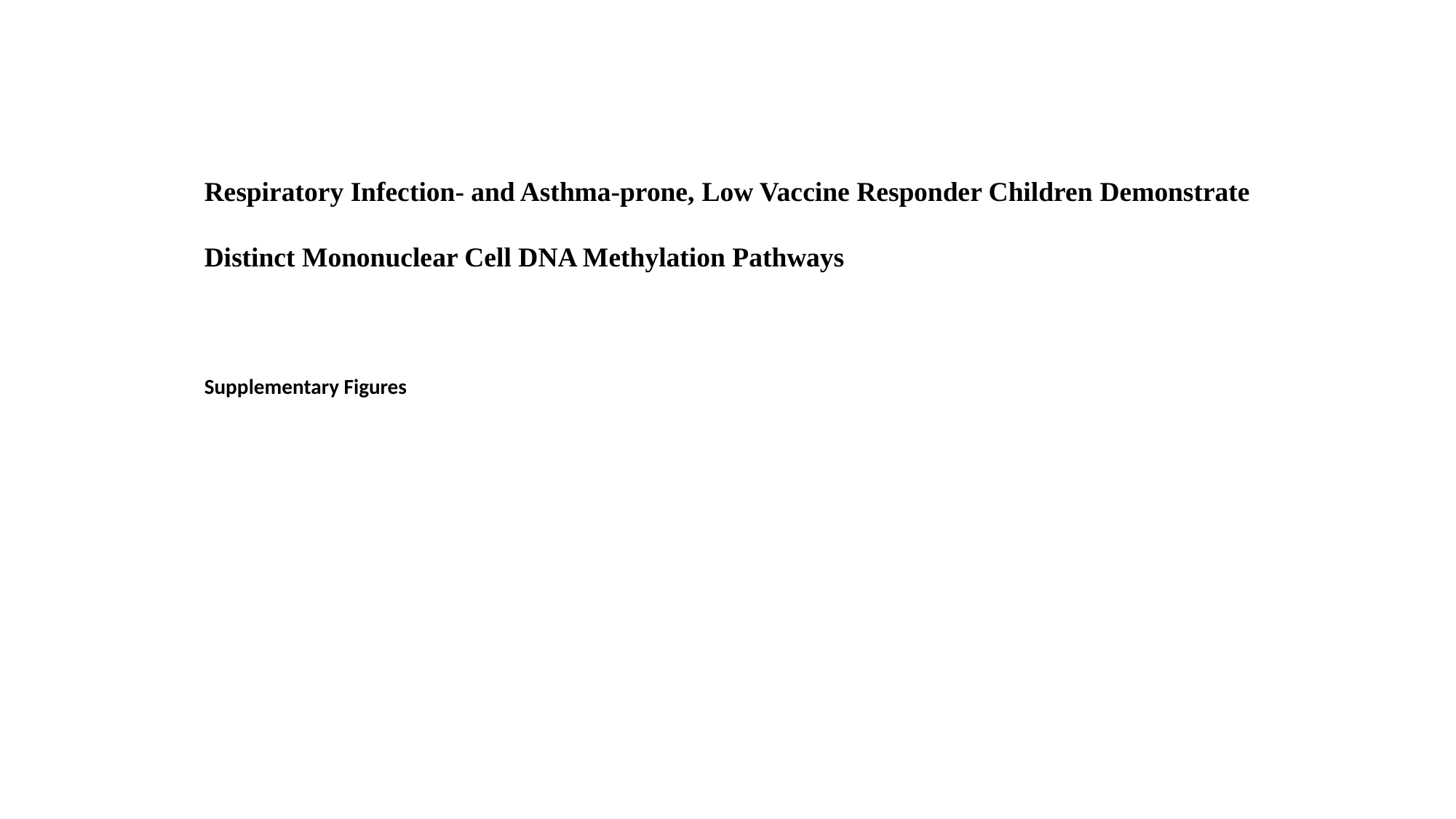

Respiratory Infection- and Asthma-prone, Low Vaccine Responder Children Demonstrate Distinct Mononuclear Cell DNA Methylation Pathways
Supplementary Figures

## Slide 2
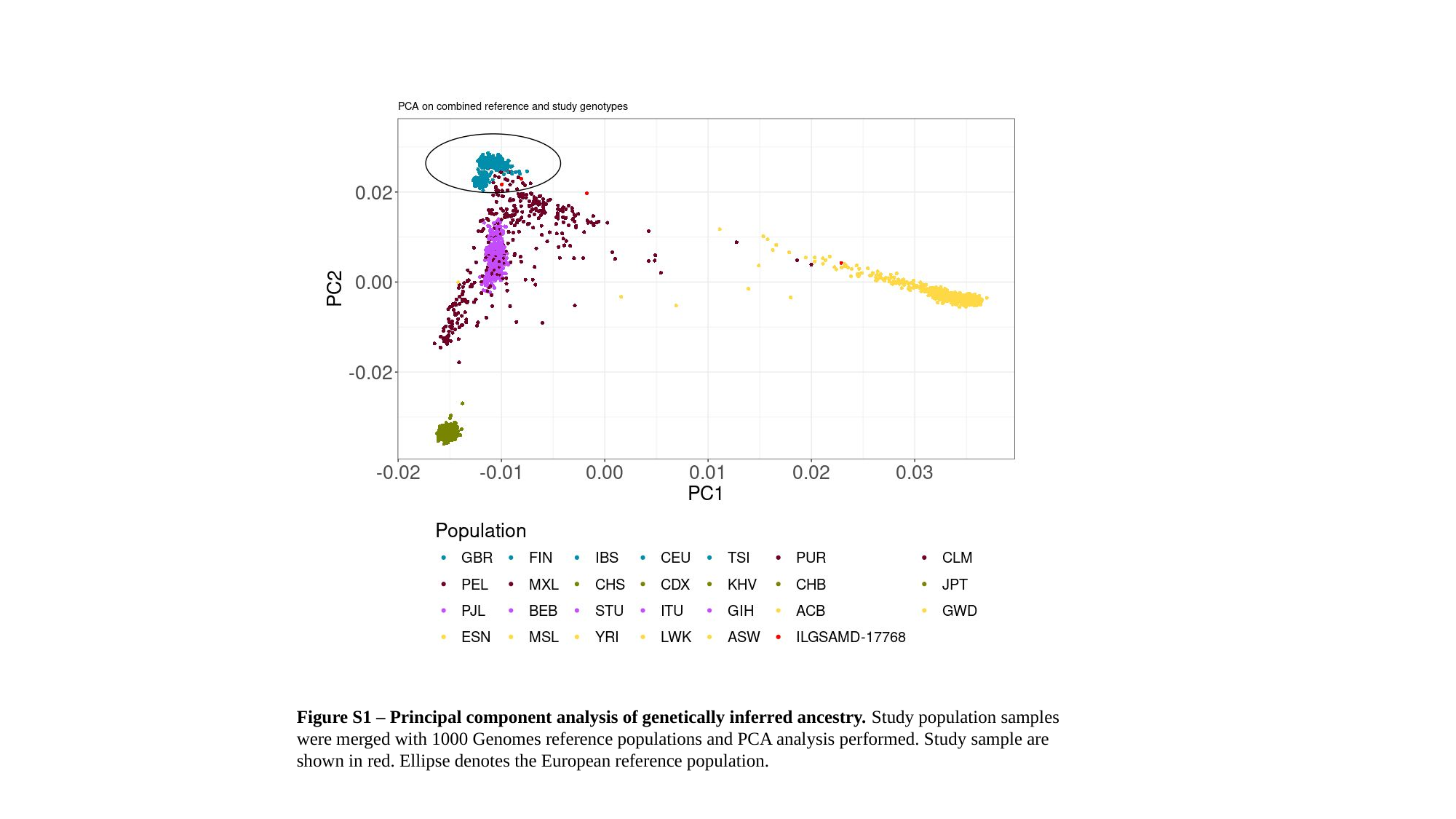

Figure S1 – Principal component analysis of genetically inferred ancestry. Study population samples were merged with 1000 Genomes reference populations and PCA analysis performed. Study sample are shown in red. Ellipse denotes the European reference population.

## Slide 3
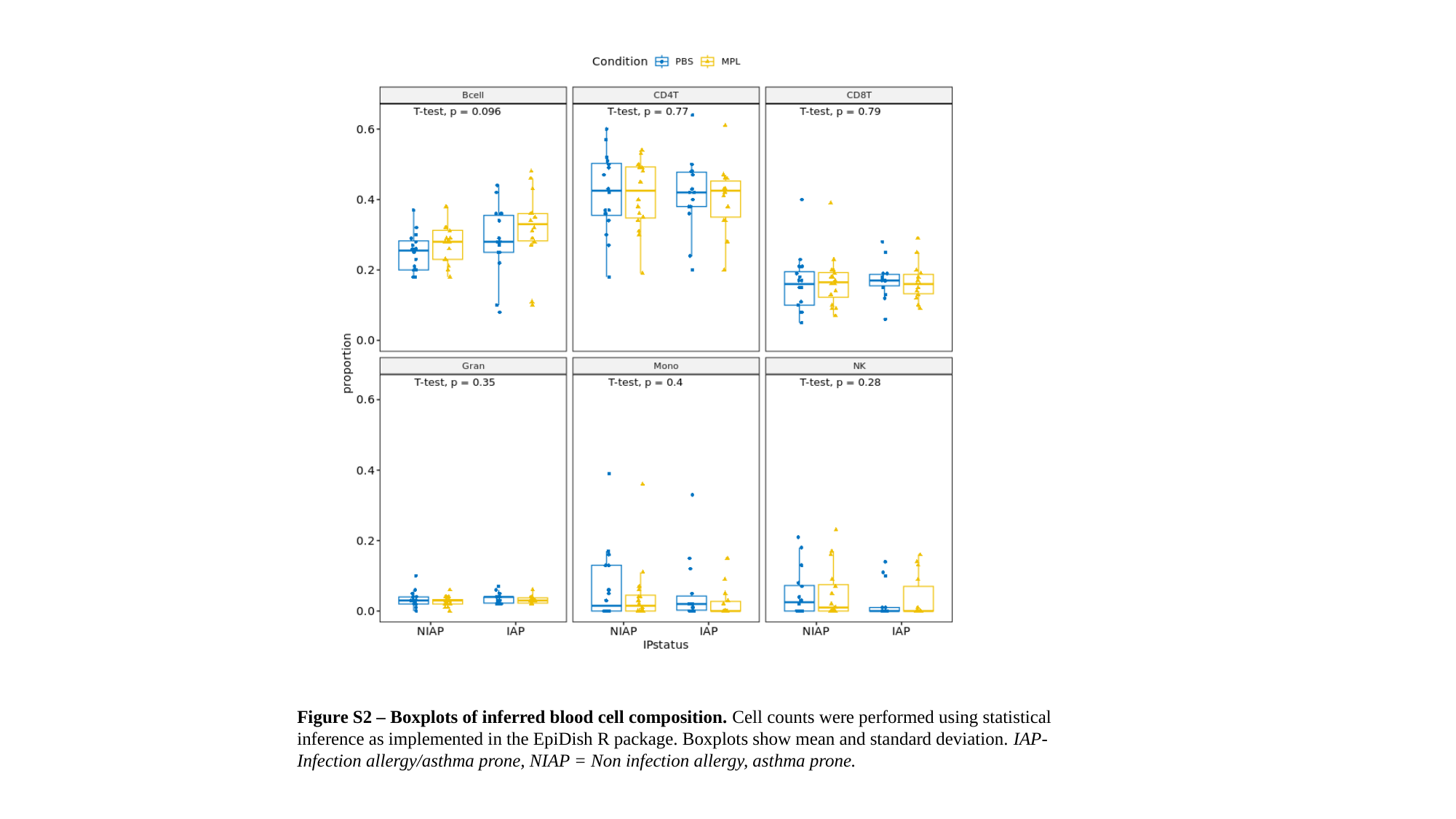

Figure S2 – Boxplots of inferred blood cell composition. Cell counts were performed using statistical inference as implemented in the EpiDish R package. Boxplots show mean and standard deviation. IAP- Infection allergy/asthma prone, NIAP = Non infection allergy, asthma prone.
